# Application of Cluster Analysis of Time Evolution for Magnetic Resonance Imaging -Derived Oxygen Extraction Fraction Mapping: A Promising Strategy for the Genetic Profile Prediction and Grading of Glioma

**DOI:** 10.3389/fnins.2021.736891

**Published:** 2021-10-04

**Authors:** Nanxi Shen, Shun Zhang, Junghun Cho, Shihui Li, Ju Zhang, Yan Xie, Yi Wang, Wenzhen Zhu

**Affiliations:** ^1^Department of Radiology, Tongji Hospital, Tongji Medical College, Huazhong University of Science and Technology, Wuhan, China; ^2^Department of Radiology, Weill Cornell Medical College, New York, NY, United States; ^3^Department of Biomedical Engineering, Cornell University, Ithaca, NY, United States

**Keywords:** oxygen extraction fraction (OEF), cluster analysis of time evolution, dynamic contrast-enhanced MRI (DCE-MRI), multi-parameter MRI, isocitrate dehydrogenase (IDH), O^6^-methylguanine-DNA methyltransferase (MGMT)

## Abstract

**Background:** The intratumoral heterogeneity of oxygen metabolism and angiogenesis are core hallmarks of glioma, unveiling that genetic aberrations associated with magnetic resonance imaging (MRI) phenotypes may aid in the diagnosis and treatment of glioma.

**Objective:** To explore the predictability of MRI-based oxygen extraction fraction (OEF) mapping using cluster analysis of time evolution (CAT) for genetic profiling and glioma grading.

**Methods:** Ninety-one patients with histopathologically confirmed glioma were examined with CAT for quantitative susceptibility mapping and quantitative blood oxygen level–dependent magnitude-based OEF mapping and dynamic contrast-enhanced (DCE) MRI. Imaging biomarkers, including oxygen metabolism (OEF) and angiogenesis [volume transfer constant, cerebral blood volume (CBV), and cerebral blood flow], were investigated to predict IDH mutation, O^6^-methylguanine-DNA-methyltransferase (MGMT) promoter methylation status, receptor tyrosine kinase (RTK) subgroup, and differentiation of glioblastoma (GBM) vs. lower-grade glioma (LGG). The corresponding DNA sequencing was also obtained. Results were compared with DCE-MRI using receiver operating characteristic (ROC) analysis.

**Results:** IDH1-mutated LGGs exhibited significantly lower OEF and hypoperfusion than IDH wild-type tumors (all *p* < 0.01). OEF and perfusion metrics showed a tendency toward higher values in MGMT unmethylated GBM, but only OEF retained significance (*p* = 0.01). Relative prevalence of RTK alterations was associated with increased OEF (*p* = 0.003) and perfusion values (*p* < 0.05). ROC analysis suggested OEF achieved best performance for IDH mutation detection [area under the curve (AUC) = 0.828]. None of the investigated parameters enabled prediction of MGMT status except OEF with a moderate AUC of 0.784. Predictive value for RTK subgroup was acceptable by using OEF (AUC = 0.764) and CBV (AUC = 0.754). OEF and perfusion metrics demonstrated excellent performance in glioma grading. Moreover, mutational landscape revealed hypoxia or angiogenesis-relevant gene signatures were associated with specific imaging phenotypes.

**Conclusion:** CAT for MRI-based OEF mapping is a promising technology for oxygen measurement and along with perfusion MRI can predict genetic profiles and tumor grade in a non-invasive and clinically relevant manner.

**Clinical Impact:** Physiological imaging provides an *in vivo* portrait of genetic alterations in glioma and offers a potential strategy for non-invasively selecting patients for individualized therapies.

## Introduction

Reprogramming oxygen metabolism and inducing angiogenesis are counted among the hallmarks of cancer (Hanahan and Weinberg, 2011). High-grade gliomas, such as anaplastic gliomas and glioblastomas (GBMs), account for the majority of malignant brain tumors in adults and have the characteristics of extensive hypoxia, high vascularization, and heterogeneity (Kaur et al., 2005; Hardee and Zagzag, 2012). Importantly, early identification of distinct genetic profiles such as IDH and O^6^-methylguanine-DNA-methyltransferase (MGMT) in combination with relevant receptor tyrosine kinases (RTKs) highlights the mutational profile of oxygen metabolism or angiogenesis, thus enabling early therapeutic intervention in patients (Chahal et al., 2010; Gluck et al., 2015; Kickingereder et al., 2015). Gliomas with IDH1 mutation were found to have significantly reduced hypoxia-inducible factor 1α (HIF-1α) and decreased neovascularization (Semenza, 2003). Methylation status of the MGMT promoter is also involved in carcinogenesis, as is highlighted by the association with angiogenic profile in GBM (Chahal et al., 2010). Additionally, accumulating evidence suggests that dynamic changes in coactivated RTK pathway involved in GBM may account for the oncogenic processes where tumor hypoxia and angiogenesis frequently coexist (Gluck et al., 2015).

Although molecular imaging probes under development for positron emission tomography and other imaging modalities aim to more directly evaluate tumor oxygenation, these methods are limited by the need of (i) specialized imaging agents with high costs and (ii) complex physiologic assumptions in data interpretation (la Fougere et al., 2011; Bulte et al., 2012; Paech et al., 2020). Moreover, this information currently confronts tissue sampling errors, as a single specimen/sample might not reflect the presence of the mutation in such a heterogeneous tumor. Consequently, alternative non-invasive magnetic resonance imaging (MRI) sequences to predict genetic profiles via its reliable oxygen information are urgently needed.

Nowadays, a novel developed approach, cluster analysis of time evolution (CAT), for quantitative susceptibility mapping and quantitative blood oxygen level–dependent magnitude (QSM + qBOLD or QQ)–based oxygen extraction fraction (OEF) mapping, might have the potential to cope with this problem (Cho et al., 2018, 2020b). The robustness of QQ-based OEF mapping has been substantially improved by introduction of an unsupervised machine learning method, CAT, which may enable clinically practical evaluation of oxygen information without vascular challenges. Because of the relative simplicity of the susceptibility measurement and an acceptable time requirement, CAT for QQ represents a non-invasive measure of quantitative OEF that meets the immediate applicability in clinical practice (Cho et al., 2020a,b). Furthermore, dynamic contrast-enhanced (DCE) MRI has long been clinically used to investigate tumor angiogenesis and plays a pivotal role for characterization of tumor microvessel proliferation and permeability, cerebral blood flow (CBF), or cerebral blood volume (CBV) (Jensen et al., 2014; Arevalo-Perez et al., 2015).

The purpose of this study was to investigate the diagnostic performance of CAT for QQ-based OEF mapping and apply DCE-MRI to predict not only the molecular parameters such as IDH1 mutation and MGMT promoter methylation status in glioma but also differentiation of World Health Organization (WHO) grade II/III lower-grade glioma (LGG) vs. WHO grade IV GBM. Impressively, we also tried to highlight the association of physiological MRI and molecular stratification via the combination of RTK genetic aberrations rather than assessing the status of individual mutations, potentially providing new therapeutic opportunities against these deadly brain tumors. Altogether, we hypothesized that CAT for QQ-based OEF mapping might be a potential method to specifically quantify oxygen metabolism in glioma, this method in combination with characterization of tumor angiogenesis assessed by DCE-MRI can potentially be used as an imaging biomarker to non-invasively identify genetic profiles in the preoperative workup of glioma patients. The established imaging signatures are derived from routine clinically acquired MRI, and therefore, it is easily translatable to the clinic.

## Materials and Methods

### Patient Selection

This retrospective study was performed with approval of the local institutional review board, and written informed consent was waived. Between July 2016 and December 2019, 138 consecutive patients with suspected primary gliomas were enrolled in this study. Forty-seven patients (34%) were excluded because of the following criteria: (a) diagnosis other than glioma (21 patients), (b) recurrent glioma undergoing therapy (12 patients), and (c) insufficient data quality, in the form of patient motion during MRI (three patients with severe neurological deficits) or poor contrast material injection (11 patients). A total of 91 pathologically confirmed gliomas were identified in our analysis. Detailed patient characteristics are further profiled in Table 1.

**TABLE 1:** Patient demographics and genetic information.

**Parameter**	**All patients**	**Patients with LGG**	**Patients with GBM**
**WHO tumor grade**	91	48 (52.7%)	43 (47.2%)
Mean age (y)*	47.1 ± 12.6	44.3 ± 11.7	50.3 ± 12.9
No. of women	45 (49.4%)	28 (58.3%)	17 (39.5%)
**IDH gene status**			
Mutated	38 (41.7%)	31 (64.5%)	7 (16.2%)
Wild type	48 (52.7%)	15 (31.2%)	33 (76.7%)
NA	5 (5.4%)	2 (4.1%)	3 (6.9%)
**MGMT promoter methylation**			
Methylated	34 (37.3%)	22 (45.8%)	12 (27.9%)
Unmethylated	55 (60.4%)	26 (54.1%)	29 (67.4%)
NA	2 (2.1%)	0 (0)	2 (4.6%)
**RTKs alterations (amplification, mutation)**			
No. of patients	53 (58.2%)	26 (49.0%)	27 (50.9%)
EGFR	28 (52.8%)	7 (26.9%)	21 (77.8%)
PDGFRA	8 (15.1%)	2 (7.7%)	6 (22.2%)
MET	9 (17.0%)	4 (15.4%)	5 (18.5%)
VEGFR2	7 (13.2%)	3 (11.5%)	4 (14.8%)

*Unless otherwise indicated, data are number of patients, with percentages in parentheses. ^∗^Data are means ± standard deviations.*

*NA, not applicable; LGG, lower-grade glioma; WHO, World Health Organization; IDH, isocitrate dehydrogenase; MGMT, O^6^-methylguanine-DNA-methyltransferase; RTKs, receptor tyrosine kinases; CNV, copy number variation.*

### Magnetic Resonance Imaging Acquisition and Data Analysis

Images including conventional MRI scans, OEF images, and DCE perfusion images were acquired during clinical workup on a 3.0-T MR scanner (Discovery 750, GE Healthcare, United States). All images were coregistered to the three-dimensional (3D) T1-BRAVO images, using FMRIB’s Linear Image Registration Tool algorithm (FSL).^1^ Details on MRI acquisition parameters and the complete analysis workflow (performed by Drs. Shen and Xie, with 8 and 5 years of experience in brain tumor image processing and interpretation of glioma imaging data, respectively) are outlined in Supplementary Methods 1.

### Magnetic Resonance Imaging-Based Oxygen Extraction Fraction Mapping Post-processing

To achieve OEF = 1 − *Y*/*Y*_*a*_, the following steps were used for MRI-based OEF postprocessing. (a) QSM images were reconstructed from multi-echo gradient echo (mGRE) data using a fully automated zero-referenced morphology-enabled dipole inversion method as described previously (Liu et al., 2018). QSM model was then generated from the decomposed susceptibility sources by using the following equation:


$$\chi_{\text{QSM}}\,\left(Y,\nu ,\chi_{\text{nb}}\right)=\left[\frac{\chi_{\text{ba}}}{\alpha }+\Psi_{\text{Hb}}\cdot △\,\chi_{\text{Hb}}\cdot \left(-\text{Y}+\frac{1-\left(1-\alpha \right)\cdot {\text{Y}}_{\text{a}}}{\alpha }\right)\right]$$

(1) $$\cdot \nu +\left(1-\frac{\nu }{\alpha }\right)\cdot \chi_{\text{nb}}$$

where α is the vein volume fraction assumed to be constant (0.77), Ψ_Hb_ is the hemoglobin volume fraction (0.0909 for tissue and 0.1197 for vein), and △χ_Hb_ is the susceptibility difference between deoxyhemoglobin and oxyhemoglobin (12,522 ppb). (b) The qBOLD model of |*s*_*j*_| based on a voxel spread function method (Cho et al., 2018) was fitted into the following equation:


$$\left|s_{j}\right|=F_{\text{qBOLD}}\,\left(Y,\nu ,\chi_{\text{nb}},{\text{s}}^{0},R_{2},j\,△\,\text{TE}\right)={\text{s}}^{0}\,{\text{e}}^{-R_{2}\cdot \text{j}\,△\,\text{TE}}$$

(2) $${\text{e}}^{-\nu \,\text{f}\,\left(\delta_{\omega }\,\left(Y,\chi_{\text{nb}}\right)\cdot \text{j}\,△\,\text{TE}\right)}\,g\,\left(\text{j}\,△\,\text{TE}\right)$$

(3) $$\delta_{\omega }\,\left(Y,\chi_{\text{nb}}\right)=\frac{1}{3}\cdot \gamma \cdot {\text{B}}_{0}\cdot \left[\text{Hct}\cdot △\,\chi_{0}\cdot \left(1-Y\right)+\chi_{\text{ba}}-\chi_{\text{nb}}\right]$$

where γ is the gyromagnetic ratio (267.513 MHz/T), *B_0* is the main magnetic field (3.0T), Hct is the hematocrit (0.357), △χ_0_is susceptibility difference between fully oxygenated and fully deoxygenated blood [4π × 0.27 ppm (Yablonskiy and Haacke, 1994)], *Y* is the oxygenation, χ_ba_ is the purely oxygenated blood susceptibility (−108.3 ppb), χ_nb_ is the non-blood susceptibility, ν is the vein blood volume fraction, and *g* accounts for the macroscopic contributions due to voxel sensitivity function. (c) The QSM model of the phase analysis and the qBOLD model of the magnitude analysis were combined by using a denoising regularization R (Cho et al., 2018):


(4) $${\text{argmin}}_{Y,\nu ,\chi_{\text{nb}},{\text{s}}^{0},{\text{R}}_{2}}\,\sum_{\text{j}}{||\left|s_{j}\right|-F_{\text{qBOLD}}\,\left(Y,\nu ,\chi_{\text{nb}},{\text{s}}^{0},R_{2},j\,△\,\text{TE}\right)||}_{2}^{2}+\text{w}\,{||\chi -\chi_{\text{QSM}}\,\left(Y,\nu ,\chi_{\text{nb}}\right)||}_{2}^{2}+\lambda \,\text{R}\,\left(Y,\nu ,\chi_{\text{nb}},{\text{s}}^{0},R_{2}\right)$$

(d) A CAT method, where voxels with similar mGRE magnitude time evolutions were grouped into a cluster, was applied to improve the effective signal-to-noise ratio (SNR) for QSM + qBOLD model (Cho et al., 2020b).

### Histological Examination

All diagnoses were histopathologically proven after surgical resection or tumor biopsy, according to the 2016 WHO classification of CNS tumors, by neuropathologists who were blinded to the MRI data. Genomic sequencing analysis was performed based on tissue availability. IDH1 mutation status was determined by using next-generation sequencing and/or immunohistochemistry (IHC). MGMT promoter methylation status (methylated vs. unmethylated) was detected by using MGMT pyrosequencing from the PyroMark Q24 sequencer. In order to further characterize genetic heterogeneity potentially driving oxygen metabolism or angiogenesis in glioma, we examined the association of established preoperative MRI parameters with coactivated RTKs based on somatic genomic alterations and hallmark copy number variation of glioma as described previously (Supplementary Methods 2; Stommel et al., 2007; Nakada et al., 2020). We also performed IHC analysis of Ki67 proteins routinely analyzed in current clinical practice, using a standard immunohistochemical staining procedure (*n* = 81).

### Statistical Analysis

Normal distribution of the parameters was tested by Shapiro–Wilk test (Razali and Wah, 2011; Das and Rahmatullah Imon, 2016). Data normality was also checked and reported by using Kolmogorov–Smirnov test. Detailed results for testing normality are shown in Supplementary Table 1 and Supplementary Figure 1. In general, the majority of the results of the two methods were consistent for testing the normality. The Shapiro–Wilk test provides greater power than the Kolmogorov–Smirnov test (even with its Lilliefors correction). Additionally, the Shapiro–Wilk test is more appropriate for small sample sizes (N ≤ 50), but it can also be validly applied with large sample sizes. For these reasons, the Shapiro–Wilk test was used for assessing data normality in our study. Then, the subgroups of participants with LGG and GBM, molecular parameters [IDH1 (mutation vs. wild-type), MGMT (methylated vs. unmethylated), activation of RTKs subgroup (RTK vs. non-RTK)] were compared by using Student *t*-test (for normally distributed data) or Wilcoxon rank-sum test (for non-normally distributed data). There is evidence that the most investigated parameters are non-normally distributed based on the Shapiro–Wilk test (Supplementary Table 1 and Supplementary Figure 1). The DeLong et al. (1988) procedure is commonly used to non-parametrically test the hypothesis of the equality of the areas under the curve (AUCs) and performs well regardless of the variable distribution. Thus, non-parametric receiver operating characteristic (ROC) AUC testing based on the Delong approach was performed to assess the predictive performance for grading and molecular detection. The cutoff values were selected by using the maximized values of the Youden indexes. Then, the sensitivity and specificity at the threshold values for each parameter were determined to evaluate the diagnostic performance. The statistical significance of the single AUC was also calculated to test the null hypothesis that the AUC really equals 0.50. The correlations between MRI biomarkers and the Ki67 expression were evaluated with Pearson correlation. For each outcome, we calculated the false discovery rate using the Benjamini–Hochberg method to account for multiple hypothesis tests (Benjamini and Hochberg, 1995). *p* < 0.05 was considered statistically significant. Analyses were performed using R software v3.6.1.

## Results

### Predictability of IDH1 Mutation Status

**FIGURE 1:**
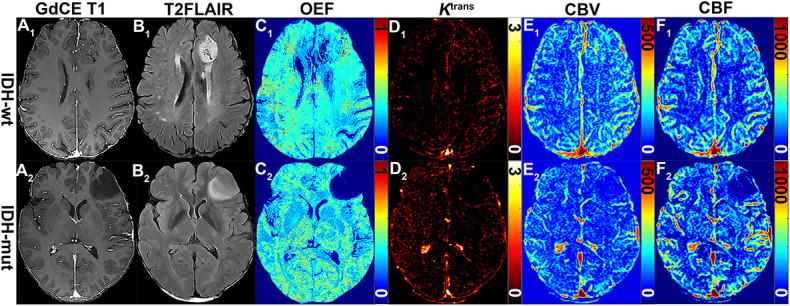
MRI-based OEF and DCE perfusion images in representative glioma patients with and without IDH mutation. Two exemplary patients with IDH1-wt (astrocytoma II; **A_1_–F_1_)** and IDH1-mut (astrocytoma II; **A_2_–F_2_)** lower-grade glioma shown: **(A_1_)** gadolinium contrast-enhanced (GdCE) 3DT1BRAVO, **(B_1_)** T2-FLAIR, **(C_1_)** QQ-based OEF maps by CAT, DCE-MRI with separated *K*^*trans*^ **(D_1_)**, CBV **(E_1_)**, and CBF **(F_1_)** maps. OEF map **(C_1_)** allows to delineate significantly inhomogeneous hyperintensity in the contrast-enhanced region of the IDH1 wild-type astrocytoma; DCE-derived maps **(D_1_–F_1_)** clearly show the area of hyperperfusion and high permeability in the IDH1 wild-type astrocytoma.

**FIGURE 2:**
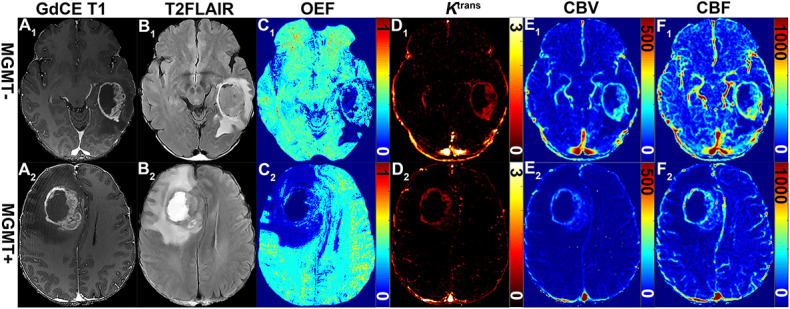
Representative MRI-based OEF and DCE perfusion images of glioma patients with and without methylated MGMT promoter. Two GBM patients with unmethylated (51-year-old woman with MGMT^–^: **A_1_–F_1_)** and methylated (58-year-old man MGMT^+^: **A_2_–F_2_)** promoter shown: **(A_1_)** GDCE 3D T1BRAVO, **(B_1_)** T2-FLAIR, **(C_1_)** QQ-based OEF maps with CAT, DCE-MRI with separated *K*^*trans*^ **(D_1_)**, CBV **(E_1_)**, and CBF **(F_1_)** maps. OEF map **(C_1_)** allows to identify a focal area of higher values within the lesion for patients with unmethylated MGMT promoter; DCE-derived maps **(D_1_–F_1_)** show a tendency toward higher signal intensities.

**FIGURE 3:**
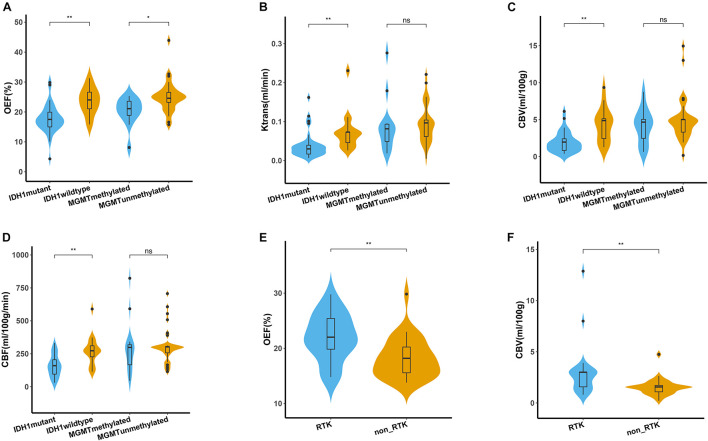
Quantitative comparison of oxygen metabolism and vascular metrics of genetic profiles. **(A–D)** Comparison of OEF, *K*^*trans*^, CBV, and CBF measurements between IDH1 mutant and wild type in lower-grade glioma (left panel), as well as between MGMT methylated and unmethylated glioblastoma (right panel). Comparison of OEF **(E)** and CBV **(F)** values between RTK and non-RTK subgroup. The violin plot marked with asterisk (*) indicates that the values were significantly different between the groups (*P < 0.05; **P < 0.01).

The representative MRI-based OEF and DCE perfusion images are shown in Figures 1, 2. For oxygen metabolism of all patients with LGG, OEF was found to be remarkably decreased (*p* = 0.001; Figure 3A) in IDH1 mutation than in IDH1 wild-type lesions. OEF did not enable IDH1 status detection in patients with GBM (*p* = 0.645; Table 2).

**TABLE 2:** Comparison of biomarkers for subgroups of IDH mutation status.

**Patients with LGG (WHO II and III)**			
**Parameter**	**IDH mut (mean ± SD)**	**IDH wt (mean ± SD)**	**FDR-adjusted *p*-value**
OEF (%)	17.67 ± 4.98	23.71 ± 5.50	**0.001** ^†^
*K*^*trans*^ (mL/min)	0.04 ± 0.03	0.08 ± 0.05	**0.004** ^‡^
CBV (mL/100 g)	1.97 ± 1.40	4.35 ± 2.33	**0.002** ^‡^
CBF (mL/100 g per min)	159.90 ± 83.66	276.34 ± 116.83	**0.006** ^‡^

**Patients with GBM (WHO IV)**			
**Parameter**	**IDH mut (mean ± SD)**	**IDH wt (mean ± SD)**	**FDR-adjusted *p*-value**

OEF (%)	23.39 ± 6.77	23.69 ± 5.52	0.645^‡^
*K*^*trans*^ (mL/min)	0.08 ± 0.05	0.10 ± 0.06	0.494^‡^
CBV (mL/100 g)	3.79 ± 2.14	5.12 ± 2.97	0.420^‡^
CBF (mL/100 g per min)	285.85 ± 63.34	321.46 ± 173.86	0.707^‡^

*OEF, oxygen extraction fraction; K^*trans*^, volume transfer constant; CBV, cerebral blood volume; CBF, cerebral blood flow.*

*p-values were calculated using Student t-test****^†^*** *or Wilcoxon rank-sum test****^‡^*** *with false discovery rate (FDR) correction (<0.05). Statistically significant p-values are highlighted in bold font.*

For angiogenesis of all patients with LGG, markedly decreased volume transfer constant (*K*^*trans*^), CBV, and CBF in IDH1 mutation were found compared with IDH1 wild-type tumors (all *p* < 0.01; Figures 3B–D). None of the investigated perfusion metrics allowed for differentiation of IDH1 gene mutation status in patients with GBM (*p* > 0.05).

### Predictability of O^6^-Methylguanine-DNA Methyltransferase Promoter Methylation Status

GBM with MGMT promoter methylation showed predominantly decreased OEF compared with unmethylated tumors (*p* = 0.01; Figure 3A). LGG demonstrated no significant differences in OEF regarding MGMT status (*p* = 0.408; Table 3).

**TABLE 3:** Comparison of biomarkers for subgroups of MGMT status.

**Patients with LGG (WHO II and III)**			
**Parameter**	**MGMT** ^+^ **(mean ± SD)**	**MGMT** ^–^ **(mean ± SD)**	**FDR-adjusted *p-*value**
OEF (%)	18.99 ± 5.65	20.70 ± 5.68	0.408^†^
*K*^*trans*^ (mL/min)	0.03 ± 0.01	0.06 ± 0.05	0.080^‡^
CBV (mL/100 g)	1.96 ± 1.05	3.27 ± 2.50	0.200^‡^
CBF (mL/100 g per min)	161.37 ± 75.27	217.20 ± 130.81	0.225^‡^

**Patients with GBM (WHO IV)**			
**Parameter**	**MGMT^+^(mean ± SD)**	**MGMT** ^–^ **(mean ± SD)**	**FDR-adjusted *p*-value**

OEF (%)	20.19 ± 4.74	25.17 ± 5.35	**0.01** ^‡^
*K*^*trans*^ (mL/min)	0.09 ± 0.07	0.10 ± 0.05	0.397^‡^
CBV (mL/100 g)	4.17 ± 2.37	5.09 ± 3.04	0.420^‡^
CBF (mL/100 g per min)	308.45 ± 213.63	312.88 ± 136.05	0.585^‡^

*OEF, oxygen extraction fraction; K^*trans*^, volume transfer constant, CBV, cerebral blood volume, CBF, cerebral blood flow.*

*p-values were calculated using Student t-test****^†^*** *or Wilcoxon rank-sum test****^‡^*** *with false discovery rate (FDR) correction (<0.05). Statistically significant p-values are highlighted in bold.*

Perfusion analysis showed a tendency toward higher values in patients with unmethylated MGMT promoter but did not allow for significant differentiation of methylated vs. unmethylated gliomas (*p* > 0.05; Figures 3B–D).

### Predictability of Receptor Tyrosine Kinase Aberrations in a Subset of Patients With Next-Generation Sequencing

The association of each imaging parameter and the corresponding RTK aberrations demonstrated a significant positive relationship between RTK subgroup and increased OEF (*p* = 0.003, Figure 3E and Table 4). Perfusion metrics (CBV and CBF) showed a tendency toward higher values in the RTK subgroup (*p* = 0.004 and *p* = 0.011, respectively, Figure 3F).

**TABLE 4:** Comparison of biomarkers for RTK subgroup.

**Parameter**	**RTK (mean ± SD)**	**Non-RTK (mean ± SD)**	**FDR-adjusted *p*-value**
OEF (%)	22.021 ± 4.191	18.191 ± 3.635	**0.003** ^†^
*K*^*trans*^ (mL/min)	0.058 ± 0.037	0.047 ± 0.031	0.137^‡^
CBV (mL/100 g)	3.003 ± 2.562	1.563 ± 0.816	**0.004** ^‡^
CBF (mL/100 g per min)	201.754 ± 104.509	140.769 ± 56.485	**0.011** ^‡^

*OEF, oxygen extraction fraction; K^*trans*^, volume transfer constant; CBV, cerebral blood volume; CBF, cerebral blood flow; RTK, receptor tyrosine kinase.*

*p-values were calculated using Student t-test^†^ or Wilcoxon rank-sum test^‡^ with false FDR correction (<0.05). Statistically significant p-values are highlighted in bold.*

### Predictability of World Health Organization Tumor Grade

When comparing imaging biomarkers in tumor cores between WHO grades, OEF demonstrated significant difference between LGG and GBM (*p* < 0.0001; Table 5). Perfusion analysis in patients with GBM showed areas with increased *K*^*trans*^, CBV, and CBF when compared with LGG (*p* < 0.0001 for each).

**TABLE 5:** Comparison of biomarkers for WHO tumor grades.

**Parameter**	**LGG (mean ± SD)**	**GBM (mean ± SD)**	**FDR-adjusted *p*-value**
OEF (%)	18.48 ± 4.85	24.17 ± 4.86	**<0.0001** ^‡^
*K*^*trans*^ (mL/min)	0.04 ± 0.02	0.09 ± 0.05	**<0.0001** ^‡^
CBV (mL/100 g)	2.11 ± 1.55	5.07 ± 4.17	**<0.0001** ^‡^
CBF (mL/100 g per min)	164.80 ± 101.10	314.10 ± 172.00	**<0.0001** ^‡^

*OEF, oxygen extraction fraction; K^*trans*^, volume transfer constant; CBV, cerebral blood volume; CBF, cerebral blood flow; LGG, lower-grade glioma; GBM, glioblastoma.*

*p-values were calculated using Wilcoxon rank-sum test^‡^ with false discovery rate (FDR) correction (<0.05). Statistically significant p-values are highlighted in bold.*

### Diagnostic Performance of Imaging Biomarkers

**FIGURE 4:**
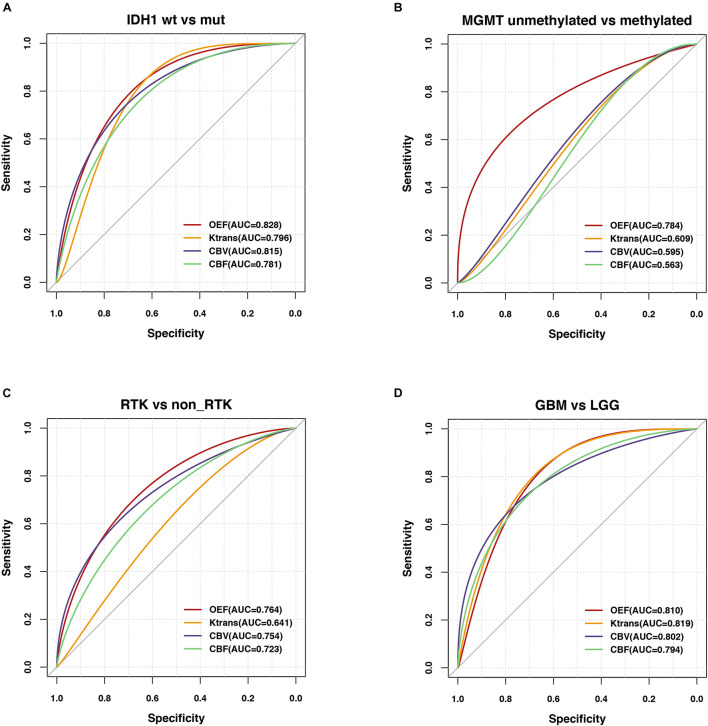
Predictability of genetic profiles and WHO tumor grade in glioma patients. ROC analyses performed for OEF and vascular metrics in order to assess the contrast ability to predict **(A)** IDH mutation status in LGG (IDH_1_–R132H mutation vs. wild type), **(B)** MGMT promoter methylation status in GBM (methylated vs. unmethylated), **(C)** RTK subgroup in a subset of patients with NGS, and **(D)** WHO tumor grade (GBM vs. LGG).

Regarding classification of molecular profiling for IDH1 mutation status, best test performance was achieved using OEF values with sensitivity and specificity of 74.20 and 86.67%, respectively, at a threshold of 0.196 of all imaging biomarkers in LGG (AUC = 0.828; *p* < 0.001; Figure 4A). Results of perfusion ROC analysis were good to excellent, albeit with slightly inferior performance (the AUCs of these parameters ranged from 0.781 to 0.815; *p* < 0.01).

Regarding MGMT promoter methylation status classification in patients with GBM, only OEF allowed for significant prediction of MGMT promoter methylation yielding an AUC of 0.784 (*p* = 0.005; Figure 4B).

In comparison of diagnostic abilities of imaging biomarkers, OEF, and perfusion metrics, best classification of the RTK subgroup was achieved using the OEF values with sensitivity and specificity of 83.33 and 72.41%, respectively, at a cutoff value of 19.28 (AUC = 0.764; *p* = 0.001). CBV also exhibited moderate performance in identification of RTKs, yielding an AUC of 0.754 (*p* = 0.002; Figure 4C).

The differentiation ability of LGG and GBM had a range of AUCs from 0.794 to 0.819 (*p* < 0.0001); reliable prediction of WHO tumor grade was possible using the OEF metric, yielding areas under the ROC curve of 0.810 (*p* < 0.0001; Figure 4D), with a sensitivity and specificity of 76.74 and 79.17%, respectively. The results of all ROC curve analyses are provided in Supplementary Table 2.

### The Mutational Landscape in the Subset of Patients With Next-Generation Sequencing

**FIGURE 5:**
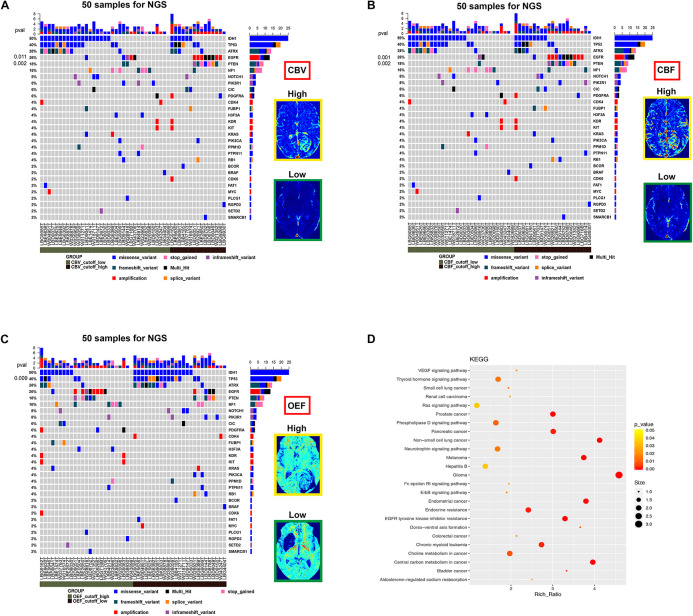
The mutational landscape and biological signatures for 50 tumor samples with NGS, 49 of which harbored genetic alterations. **(A–C)** Samples from patients have been stratified according to the cutoff value of imaging parameters (CBV, CBF, and OEF) for RTK aberrations. The heat map below displays the somatic mutated genes ordered by their respective mutation frequencies. The significant genes with a *p*-value < 0.05 were labeled on the left. **(D)** Kyoto Encyclopedia of Genes and Genomes pathway analysis enriched in the RTK subgroup.

A more detailed view of molecular biology revealed that the most significantly prominent genes of epidermal growth factor receptor (EGFR) (*p* = 0.001), PTEN (*p* = 0.002), and TP53 (*p* = 0.009) between two groups (RTK vs. non-RTK) separated by the optimized thresholds of imaging parameters were identified (Figures 5A–C). These genes are closely linked to angiogenesis or tumor hypoxia (Amelio and Melino, 2015; Gluck et al., 2015; An et al., 2018). Of note is that the Kyoto Encyclopedia of Genes and Genomes pathway analysis revealed biologically interesting insights mainly enriched in cancer- and angiogenesis-related pathways including glioma, vascular endothelial growth factor (VEGF) signaling, and EGFR tyrosine kinase inhibitor resistance in the RTK subgroup (*p* < 0.05; Figure 5D).

### Correlation Between Magnetic Resonance Imaging Measurements With Immunohistochemistry Results

**FIGURE 6:**
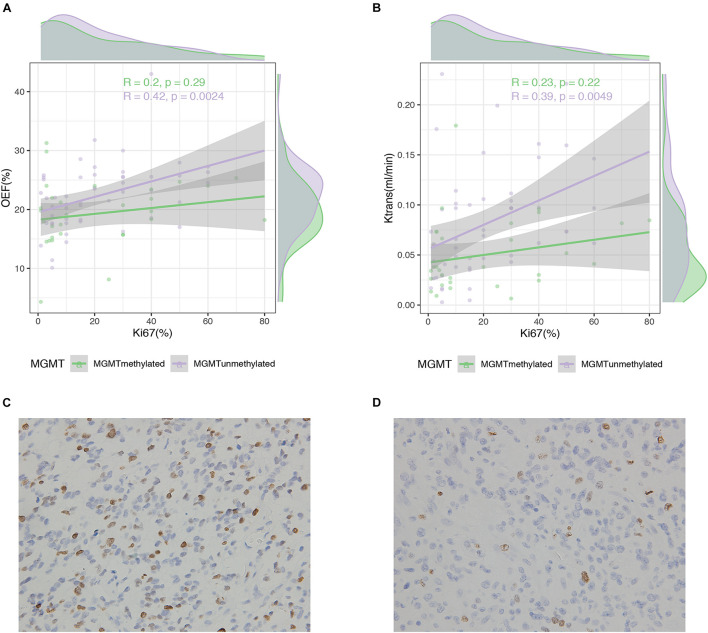
Correlation between MRI measures and Ki67 expression in glioma patients. Scatterplot with linear regression line shows a weak to moderate association between MRI measures of OEF **(A)** and K^*trans*^ **(B)** with Ki67 expression in MGMT promoter methylated and unmethylated gliomas followed by Ki-67 immunohistochemistry on the lower panel **(C,D)**.

Specifically, histological results in our study also indicated that OEF values (*r* = 0.42, *p* = 0.002) or less relevant *K*^*trans*^ values (*r* = 0.39, *p* = 0.005) were positively correlated with the cell cycle marker Ki67, but only in MGMT unmethylated gliomas (Figure 6). Apart from that, Ki67 in IDH mutant and/or wild-type gliomas did not correlate with OEF or any perfusion metrics (*p* > 0.05).

## Discussion

In this study, we investigated CAT for QQ-based oxygenation extraction fraction mapping; meanwhile, DCE-MRI was explored to demonstrate the non-invasive predictability of genetic profiles and WHO tumor grade in glioma patients. CAT for QQ-based OEF mapping that is respiratory challenge-free is a clinically compatible and potentially valuable imaging technique for oxygenation mapping. The current study revealed the feasibility of CAT for QQ-based OEF mapping, along with characterization of tumor angiogenesis assessed by using DCE-MRI, can potentially be used as non-invasive imaging biomarkers for genetic profile prediction and glioma grading in the clinical routine.

Regional OEF is an essential biomarker for investigating tissue metabolism and function in various diseases such as stroke (Derdeyn et al., 1998, 2002), cerebral tumors (Ito et al., 1982), and Alzheimer’s disease (Ishii et al., 1996). The OEF mapping is achieved via application of CAT to denoise QSM + qBOLD (QQ) estimation of OEF based on 3D multiecho gradient echo acquisition. The CAT algorithm based on unsupervised machine learning substantially improves the robustness against noise of QQ-based mapping of OEF within a few minutes. This CAT for QQ-based OEF mapping eliminates assumptions and is theoretically more accurate than other OEF mapping methods (Kudo et al., 2016; Zhang et al., 2018). Therefore, rapid OEF mapping can be included in routine clinical MRI protocols to accurately evaluate oxygen metabolism in glioma patients and, upon further validation, would help patient stratification for targeted therapy.

QQ-based OEF mapping with CAT performed markedly better in prediction of IDH1 mutation status than WHO tumor grade in this study cohort. Compared with perfusion ROC analyses, the OEF mapping yielded good prediction of IDH mutation status. Therefore, we infer that genetic status, by means of IDH1 mutation status, is more reflective than the histologic class, regarding oxygen metabolism or tumor vascularization. The latest WHO classification of CNS tumors termed gliomas of WHO grades I–III together as “lower-grade gliomas” with a great majority of IDH1 mutations and a wide range of overall survival within this group (Cancer Genome Atlas Research Network et al., 2015; Louis et al., 2016). Mutations in IDH1 gene–encoded enzymes are expected to cause widespread disturbances of cellular metabolism, including DNA hypermethylation with subsequently increased tumor metabolism and degradation of HIF, leading to downstream inhibition of vasculogenesis- and angiogenesis-related signaling (Ye et al., 2013). Hence, the investigated OEF values and vascular metrics are expected to be significantly lower in IDH1-mutated tumors, in agreement with our study findings. It is conceivable that lower OEF and perfusion values in IDH1-mutated gliomas could be due to lower proliferation rates (Bralten et al., 2011) and less angiogenesis (Kickingereder et al., 2015) compared with their wild-type counterparts.

OEF mapping allowed for prediction of MGMT promoter methylation status that is difficult to detect in perfusion MRI. Tumors with unmethylated MGMT promoter showed a trend toward hyperperfusion in our data, in agreement with previous results obtained from perfusion imaging (Choi et al., 2017; Han et al., 2018). MGMT, the gene encoding the DNA repair enzyme O^6^-methylguanine-DNA methyltransferase, is methylated in 30–40% of GBM and 80% of IDH1-mutated LGGs (Hegi et al., 2005). Unmethylated MGMT is associated with high levels of MGMT expression and consequently induces upregulation of HIF-1α, elevated hypoxia, and increased angiogenic potential (Cao et al., 2009; Pistollato et al., 2010; Jue et al., 2017). Transferred to our findings, this may explain higher OEF and perfusion in the GBMs with unmethylated MGMT promoter compared with methylated phenotype. This observation appears consistent with results from Chahal et al. (2010) showing that a direct link between MGMT expression and decreased angiogenicity and tumorigenicity of GBM cells. Most importantly, these distinguishable molecular signatures such as IDH and MGMT can be translated into distinct phenotypes and are non-invasively predictable with physiologic MRI in a clinical setting.

Interestingly, we found moderate associations of increased OEF and perfusion metrics in the designated RTK subgroup, showing a performance of imaging biomarkers to predict relevant RTKs implicated in glioma. Our finding of elevated imaging parameters in tumors may correspond to the promotion of angiogenesis and hypoxia in a subset of patients via amplification and mutational activation of RTK-encoding genes (Stommel et al., 2007). Aberrant activation of RTKs promotes increased angiogenesis through multiple downstream effectors, which mediate a variety of vascular activities, including endothelial cell proliferation, migration, and new vessel formation (Gluck et al., 2015). As to signaling interplays between RTK activity and tumor hypoxia, the activation of VEGF receptor (VEGFR) via HIF-mediated VEGF transcriptional activation and consequent accelerated tumor angiogenesis represents one of the commonly described mechanisms that confer aggressive manifestation of hypoxic tumors (Forsythe et al., 1996). Moreover, activation of the RTK EGFR is a critical pathogenetic event involved in GBMs, with amplification and mutation, which promotes angiogenesis via distinct signaling pathways. In line with the finding of elevated relative CBV in patients with EGFR amplification by Kickingereder et al. (2016), such research efforts are essential to develop targeted therapies for more personalized cancer management.

In the current study, quantitative analysis regarding WHO grade showed increased OEF, vascular permeability, and perfusion metrics in GBM compared to LGG. OEF mapping has a fair prediction ability of WHO tumor grade. The preliminary correlation between HIF-1α expression (as per IHC) and increased OEF areas (Tóth et al., 2013) might well reflect hypoxic areas, highly characterized GBM, and thus aid in the differentiation between LGG and GBM. Our findings are in accordance with generally accepted knowledge about the mechanisms of glioma-associated neovascularization and the role of oxygen metabolism in the following processes (Hardee and Zagzag, 2012). Initially, tumor cells infiltrate through the central nervous system as well as increase oxygen extraction and nutrient supplies through vascular co-option of intact native blood vessels. Involution of co-opted vessels resulted in tumor hypoxia, upregulation of proangiogenic factors, and shift toward an angiogenic phenotype (Holash et al., 1999). Interestingly, higher OEF values were most prevalent in the peripheral area, which also fits with recent observations of Pistollato et al. (2010), who demonstrated expression of hypoxia markers (HIF-1α and VEGF) in this region. Therefore, multiparametric MRI-based physiological imaging may provide a better characterization of tumor microenvironment and yield grading of gliomas in clinical routine.

There are several limitations in the current study. The numbers of patients for the GBM with mutant IDH1 gene were relatively small. Up to now, this study recruited the largest patient cohort with glioma to obtain QQ-based OEF mapping with CAT. IDH1, however, is mutated in only ∼10% of GBMs (Yan et al., 2009). A further limitation of our study is that we did not include a validation of our approach. Biological validation of the MRI-based imaging for OEF and vascular parameters is required by correlation with findings from invasive methods or other imaging modalities in independent study cohorts.

## Conclusion

CAT for QQ-based OEF mapping, along with DCE perfusion MRI, enables superior predictability of the molecular characteristics including IDH mutation, MGMT promoter methylation status and RTK subgroup, and fair performance in differentiation of GBM vs. LGG. Impressively, the imaging biomarkers provided macroscopic insights into the heterogeneity of the biological changes in oxygen metabolism or angiogenesis that is potentially driven by genetic aberrations and shed a new light onto potential patient subsets for targeted therapy. Therefore, QQ−based OEF mapping by CAT and tumor-associated angiogenesis assessed by DCE-MRI enables prediction of genetic features and glioma grading, significantly extending the existing repertoire of non-invasive imaging biomarkers used in the preoperative workup and treatment guidance for patients with glioma.

## Data Availability Statement

The original contributions presented in the study are included in the article/Supplementary Material, further inquiries can be directed to the corresponding author/s.

## Ethics Statement

The studies involving human participants were reviewed and approved by the Tongji Hospital, Tongji Medical College, Huazhong University of Science and Technology, Wuhan, China. The ethics committee waived the requirement of written informed consent for participation.

## Author Contributions

WZ and YW contributed to the conception and design of the study. NS, SZ, and JC carried out the data collection and evaluation. SL and JZ performed the statistical analyses and visualization. NS and YX wrote the manuscript. All authors critically reviewed and approved the manuscript.

## Conflict of Interest

The authors declare that the research was conducted in the absence of any commercial or financial relationships that could be construed as a potential conflict of interest.

## Publisher’s Note

All claims expressed in this article are solely those of the authors and do not necessarily represent those of their affiliated organizations, or those of the publisher, the editors and the reviewers. Any product that may be evaluated in this article, or claim that may be made by its manufacturer, is not guaranteed or endorsed by the publisher.

## Footnotes

1
http://fsl.fmrib.ox.ac.uk/fsl/fslwiki/FSL

## Abbreviations

OEF: oxygen extraction fraction
CAT: cluster analysis of time evolution
*K* ^*trans*^: volume transfer constant
CBV: cerebral blood volume
CBF: cerebral blood flow
NGS: next-generation sequencing
IDH: isocitrate dehydrogenase
MGMT: O^6^-methylguanine-DNA-methyltransferase
RTK: receptor tyrosine kinase
GBM: glioblastoma
LGG: lower-grade glioma
HIF-1 α: hypoxia-inducible factor 1 α .
