# Supplementary material for: Application of Cluster Analysis of Time Evolution for Magnetic Resonance Imaging -Derived Oxygen Extraction Fraction Mapping: A Promising Strategy for the Genetic Profile Prediction and Grading of Glioma
Source: Front Neurosci. 2021 Oct 4;15:736891. doi: 10.3389/fnins.2021.736891 (PMC8520989; doi:10.3389/fnins.2021.736891)
Supplement: Supplementary file 1 [file Data_Sheet_1.ZIP › 736891_SupMaterial/736981_SupMaterial.docx]

Supplementary Material

**Supplemental Methods S1.**

**MR Image Acquisition**

MR imaging examinations were acquired on a 3.0T clinical MR scanner (Discovery 750, GE Healthcare, USA) equipped with a standard 32-channel head coil. The conventional MR imaging protocol included the following sequences: (a) axial T1-weighted sequences (repetition time [TR] = 2992.3 ms; echo time [TE] = 23.4 ms; field of view [FOV] = 240 × 240 mm^2^; matrix = 320 × 320; slice thickness = 5 mm), (b) axial T2-weighted sequence (TR/TE = 4599/105 ms; FOV = 240 × 240 mm^2^; matrix = 320 × 224; slice thickness = 5 mm), (c) axial fluid attenuation inversion recovery (FLAIR) sequence (TR/TE = 8000/164 ms; FOV = 240 × 240 mm^2^; matrix = 256 × 256; slice thickness = 5 mm), (d) postcontrast T1-weighted 3-dimensional brain volume (3D BRAVO) sequence (TR/TE = 8.2/3.2 ms; FOV = 240 × 240 mm^2^; matrix = 256 × 256; flip angle = 12°; slice thickness = 1.2 mm).

MRI‐based oxygen extraction fraction (OEF) mapping was performed employing a custom-developed sequence based on a 3D multiecho gradient-echo sequence (3D mGRE; TR/TE= 43.2/4.5 ms, number of TEs = 8, FOV = 240 × 240 mm^2^, matrix = 416 × 320, slice thickness = 2 mm, flip angle = 20^◦^, scan time = 5 min 15 s).

Pre-contrast T1 mapping sequences with five flip angles (3, 6, 9, 12 and 15 degrees) were acquired before injection of the bolus. After a delay of six baseline acquisitions (30 seconds), DCE-MRI perfusion images were obtained using a gradient echo T1-weighted images with 40 dynamic phases. Subsequently, an intravenous injection of gadodiamide (Omniscan, GE Healthcare, Dublin, Ireland) was administrated at a rate of 3mL/sec via a power injector (at a dose of 0.1 mmol per kilogram of body weight), followed by a 30-mL saline bolus. The specific imaging parameters were as follows: TR/TE = 3.5/1.0 ms; flip angle = 12°; FOV = 240 × 240 mm^2^; matrix = 192 × 160; section thickness = 5.0 mm; and total acquisition time, 4 minutes 7 seconds. Postprocessing of dynamic contrast-enhanced MR imaging data was performed with dedicated software (OmniKinetics; GE Healthcare). Blood perfusion maps were generated by first computing the patient-specific baseline T1 maps derived from variable flip angle sequences. Then the arterial input function was manually selected by positioning a region of interest (ROI) in the superior sagittal sinus at the level of the lateral ventricles. Finally, the perfusion parametric maps of cerebral blood volume (CBV), cerebral blood flow (CBF), and pharmacokinetic *K*^trans^ parameter were calculated by using the two-compartment extended Tofts model.

**Data analysis**

The Functional Magnetic Resonance Imaging of the Brain (FMRIB) software library (FSL; http://fsl.fmrib.ox.ac.uk/fsl/fslwiki/FSL) was used for image registration. First, brain voxels were isolated by generating a binary brain mask from the T1 volume by using the brain extraction tool and transferred to all other imaging volumes (T1-weighted images, T2-weighted images, FLAIR, OEF maps, and perfusion maps) for each patient. These imaging volumes were then registered to the brain-extracted T1 volume by using the linear image registration tool with a mutual information algorithm and 6 degree of freedom transformation.

Tumor segmentation was performed semiautomatically (by Shen or Xie, with 8 and 5 years of experience in brain tumor image processing and interpretation of glioma imaging data, respectively) to select the contrast-enhanced (CE) portion of the whole tumor (on the 3D T1BRAVO images), as well as the non-enhanced FLAIR hyperintense (NE) portion (defined as FLAIR hyperintense abnormality excluding the CE and necrotic [NEC] tumor portions, that is, including both FLAIR hyperintense tumor and potentially vasogenic edema) and the NEC portion of the tumor (on the 3D BRAVO images) using a region-growing segmentation algorithm implemented in ITK-SNAP (www.itksnap.org), as described previously. For each subject, regions of contralateral normal-appearing white matter were manually segmented in contralateral white matter and used as normal controls. All regions were combined into single, whole-tumor ROI. These different ROIs were then transferred to corresponding other modalities.

**Supplemental Methods S2.**

**Analyses of RTK Gene Aberrations in Glioma Tissue Specimens**

To investigate the potential associations of imaging biomarkers with aberrations of RTKs, copy-number variations, indels, and somatic single nucleotide variations were assessed for each patient based on tissue availability. Fifty-three patients (58%) from the cohort underwent next-generation sequencing (NGS) to identify the most commonly RTK genes within their tumors. We performed DNA isolation from fresh-frozen surgical excision specimens and determined CNVs, indels, and SNVs for all tissue samples. The prepared DNA was sequenced on a Hiseq X10 platform (Illumina, San Diego, CA) obtained with a custom panel. Multiple RTKs, including epidermal growth factor receptor (EGFR), platelet-derived growth factor receptor alpha (PDGFRA), mesenchymal-epithelial transition factor (MET) and vascular endothelial growth factor receptor2 (VEGFR2) were assessed as previously described. All genetic aberrations that activate multiple RTKs were grouped together on the basis of the RTK gene aberrations involved in glioma. Accordingly, each tumor was assigned to RTK v.s. non-RTK subgroup when at least one aberrant gene was detected from the relevant RTKs by NGS. Given the clinical significance of imaging parameters of gliomas, we investigated the differences in the somatic mutations between two groups separated by thresholds of each parameter with relevant clinical implications. In combination, significant genes were identified as being affected by mutations and/or CNVs, on the basis of which we delineated the landscape of genetic alterations according to the investigated metrics. Kyoto Encyclopedia of Genes and Genomes (KEGG) pathway enrichment analyses were used to determine the related functions and pathways for the RTK subgroup.

# Supplementary Tables

**Table S1 Kolmogorov-Smirnov and Shapiro–Wilk Tests for Assessing the Normality**

| **Subgroup Kolmogorov-Smirnov test P-value*** | | | | | | **Shapiro–Wilk test P-value** | | | | |
| --- | --- | --- | --- | --- | --- | --- | --- | --- | --- | --- |
|  | **OEF** | ***K*^trans^** | **CBV** | **CBF** | **OEF** | | ***K*^trans^** | **CBV** | **CBF** |  |
| **IDH status for LGG** | | | | | | | | | | |
| IDH mutation | 0.0503 | **0.000683** | **0.0361** | 0.183 | 0.0914 | | **0.000506** | **0.0284** | **0.0336** |  |
| IDH wildtype | 0.991 | **0.0129** | 0.428 | 0.366 | 0.921 | | **0.00269** | 0.209 | 0.145 |  |
| **IDH status for GBM** | | | | | | | | | | |
| IDH mutation | 0.355 | **0.00549** | 0.757 | 0.0528 | 0.262 | | **0.0320** | 0.890 | 0.204 |  |
| IDH wildtype | **0.0122** | **0.00313** | **0.000186** | **2.34e-05** | **0.000510** | | **0.00153** | **0.00107** | **0.00542** |  |
| **MGMT status for LGG** | | | | | | | | | | |
| MGMT methylated | 0.0996 | **0.00195** | 0.151 | 0.257 | 0.153 | | **0.00932** | 0.263 | 0.0490 |  |
| MGMT unmethylated | 0.334 | **0.0265** | 0.0509 | 0.175 | 0.538 | | **0.00111** | **0.0206** | 0.0778 |  |
| **MGMT status for GBM** | | | | | | | | | | |
| MGMT methylated | 0.427 | **0.000914** | 0.353 | 0.169 | 0.0661 | | **0.00314** | 0.187 | 0.0552 |  |
| MGMT unmethylated | 0.0592 | **0.0112** | **0.000147** | **0.000145** | **0.00710** | | **0.0224** | **0.000335** | **0.0108** |  |
| **RTK subgroup** | | | | | | | | | | |
| RTK | 0.0943 | **0.0168** | **5.33e-06** | **0.000103** | 0.328 | | **0.00765** | **0.000368** | **0.00426** |  |
| non-RTK | 0.195 | 0.0519 | **0.0318** | **0.0478** | 0.376 | | **0.00274** | 0.144 | **0.00873** |  |
| **WHO Tumor Grades** | | | | | | | | | | |
| GBM | **0.0296** | **0.000242** | **0.000122** | **1.36e-05** | **0.00483** | | **0.000666** | **0.000421** | **0.00103** |  |
| LGG | 0.153 | **0.00056** | **0.00102** | **0.0322** | 0.290 | | **1.73e-05** | **0.000850** | **0.00410** |  |

Note. — OEF = oxygen extraction fraction, *K*^trans^ = volume transfer constant, CBV = cerebral blood volume, CBF = cerebral blood flow,

LGG = lower-grade glioma, GBM = glioblastoma, RTKs = receptor tyrosine kinases.

Asterisks (*) denote that one-sample Kolmogorov-Smirnov test with Lilliefors corrected. Statistically significant P values are highlighted in bold.

**Table S2 ROC Curve Analysis for OEF Mapping and DCE-MRI**

| **IDH status for LGG (IDH1-R132H wildtype vs mutation)** | | | | | | |
| --- | --- | --- | --- | --- | --- | --- |
| **parameter** | **AUC (95% CI)** | **SEN (%)** | **SPE (%)** | **Cutoff** | **P-value** |  |
| OEF (%) | 0.828(0.688,0.923) | 74.20 | 86.67 | 0.196 | **<.001*** |  |
| *K*^trans^(ml/min) | 0.796(0.651,0.900) | 77.42 | 80.00 | 0.041 | **.002*** |  |
| CBV(ml/100g) | 0.815(0.673,0.914) | 74.19 | 80.00 | 1.992 | **.001*** |  |
| CBF(ml/100g/min) | 0.781(0.634,0.889) | 73.33 | 80.65 | 259.88 | **.003*** |  |
| **IDH status for GBM (IDH1-R132H wildtype vs mutation)** | | | | | | |
| **parameter** | **AUC (95% CI)** | **SEN (%)** | **SPE (%)** | **Cutoff** | **P-value** |  |
| OEF (%) | 0.563(0.397,0.719) | 57.14 | 78.79 | 0.207 | .322 |  |
| *K*^trans^ (ml/min) | 0.597(0.431,0.749) | 85.71 | 60.61 | 0.085 | .247 |  |
| CBV(ml/100g) | 0.617(0.450,0.766) | 71.43 | 51.52 | 4.669 | .210 |  |
| CBF(ml/100g/min) | 0.548(0.383,0.705) | 85.71 | 45.45 | 287.20 | .654 |  |
| **MGMT status for LGG (methylated vs unmethylated)** | | | | | | |
| **parameter** | **AUC (95% CI)** | **SEN (%)** | **SPE (%)** | **Cutoff** | **P-value** |  |
| OEF (%) | 0.582(0.431,0.723) | 72.73 | 50.00 | 0.212 | .209 |  |
| *K*^trans^ (ml/min) | 0.671(0.521,0.800) | 86.36 | 61.54 | 0.041 | .063 |  |
| CBV(ml/100g) | 0.630(0.479,0.765) | 90.91 | 50.00 | 3.219 | .100 |  |
| CBF(ml/100g/min) | 0.622(0.471,0.758) | 72.73 | 61.54 | 175.45 | .112 |  |
| **MGMT for GBM (methylated vs unmethylated)** | | | | | | |
| **parameter** | **AUC (95% CI)** | **SEN (%)** | **SPE (%)** | **Cutoff** | **P-value** |  |
| OEF (%) | 0.784(0.628,0.897) | 83.33 | 65.52 | 0.24 | **.005*** |  |
| *K*^trans^ (ml/min) | 0.609(0.444,0.758) | 83.33 | 58.62 | 0.096 | .199 |  |
| CBV(ml/100g) | 0.595(0.391,0.798) | 75.00 | 55.17 | 4.682 | .209 |  |
| CBF(ml/100g/min) | 0.563(0.400,0.717) | 66.67 | 58.62 | 298.46 | .292 |  |
| **RTK subgroup (RTK vs non_RTK)** | | | | | | |
| **parameter** | **AUC (95% CI)** | **SEN (%)** | **SPE (%)** | **Cutoff** | **P-value** |  |
| OEF (%) | 0.764(0.627,0.869) | 83.33 | 72.41 | 19.28 | **.001*** |  |
| *K*^trans^ (ml/min) | 0.641(0.497,0.768) | 66.67 | 79.31 | 0.048 | .069 |  |
| CBV(ml/100g) | 0.754(0.617,0.862) | 58.33 | 93.10 | 2.256 | **.002*** |  |
| CBF(ml/100g/min) | 0.723(0.583,0.837) | 70.83 | 82.76 | 174.81 | **.005*** |  |
| **WHO Tumor Grades (GBM vs LGG)** | | | | | | |
| **parameter** | **AUC (95% CI)** | **SEN (%)** | **SPE (%)** | **Cutoff** | **P-value** |  |
| OEF (%) | 0.810(0.714,0.885) | 76.74 | 79.17 | 0.212 | **<.0001*** |  |
| *K*^trans^(ml/min) | 0.819(0.724,0.892) | 83.72 | 77.08 | 0.045 | **<.0001*** |  |
| CBV(ml/100g) | 0.802(0.705,0.878) | 81.40 | 72.92 | 2.297 | **<.0001*** |  |
| CBF(ml/100g/min) | 0.794(0.696,0.871) | 62.79 | 89.58 | 277.64 | **<.0001*** |  |

Note. — AUC = area under the receiver operating characteristic curve, CI = confidence interval,

Sensitivity=SEN, Specificity=SPE.

Asterisks (*) denote that they are statistically significant with false discovery rate (FDR) correction (< 0.05).

**Supplementary Figure**

**
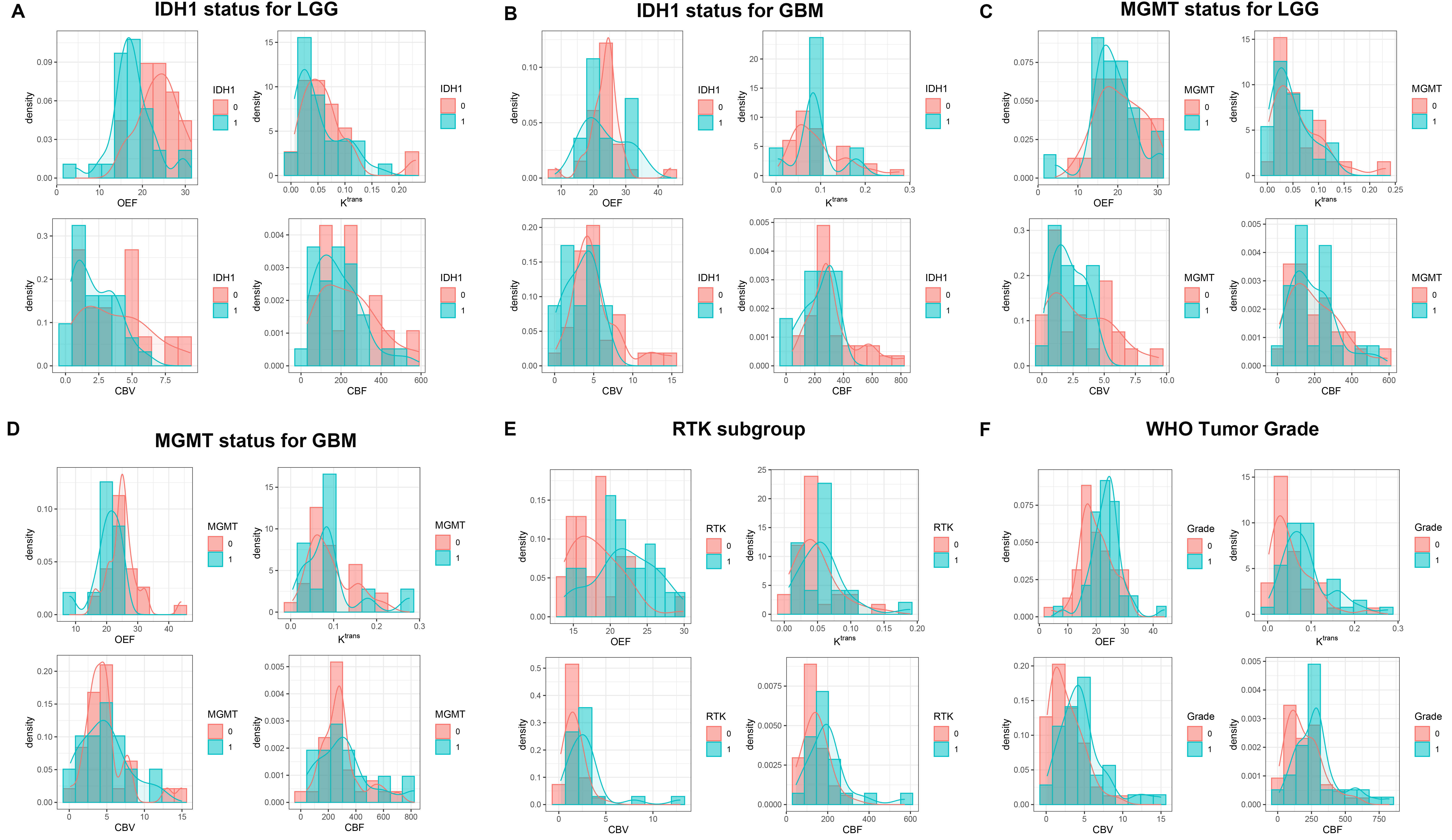
**

Figure S1 Histograms of the normal distributed data and non-normal distributed data for the investigated metrics in (A, B) IDH1 status for LGG and GBM (IDH1mutation vs wildtype), (C, D) MGMT status for LGG and GBM (MGMT methylated vs unmethylated), (E) RTK subgroup (RTK vs non-RTK), and (F) WHO Tumor Grade (GBM vs LGG).
